# Sample size planning for complex study designs: A tutorial for the mlpwr package

**DOI:** 10.3758/s13428-023-02269-0

**Published:** 2023-11-29

**Authors:** Felix Zimmer, Mirka Henninger, Rudolf Debelak

**Affiliations:** Psychological Methods, Evaluation and Statistics, Department of Psychology, University of Zurich, Binzmuehlestrasse 14, Box 27, 8050 Zurich, Switzerland

**Keywords:** Simulation, Sample size, Power analysis, Machine learning

## Abstract

A common challenge in designing empirical studies is determining an appropriate sample size. When more complex models are used, estimates of power can only be obtained using Monte Carlo simulations. In this tutorial, we introduce the R package mlpwr to perform simulation-based power analysis based on surrogate modeling. Surrogate modeling is a powerful tool in guiding the search for study design parameters that imply a desired power or meet a cost threshold (e.g., in terms of monetary cost). mlpwr can be used to search for the optimal allocation when there are multiple design parameters, e.g., when balancing the number of participants and the number of groups in multilevel modeling. At the same time, the approach can take into account the cost of each design parameter, and aims to find a cost-efficient design. We introduce the basic functionality of the package, which can be applied to a wide range of statistical models and study designs. Additionally, we provide two examples based on empirical studies for illustration: one for sample size planning when using an item response theory model, and one for assigning the number of participants and the number of countries for a study using multilevel modeling.

## Introduction

Reliable testing of scientific hypotheses requires a sufficiently large sample size. A ubiquitous challenge in empirical research is that recruiting large samples is difficult due to resource constraints (e.g., time, money, labor) or ethical constraints (e.g., inconvenience or participation risks). However, if the sample sizes are small, random noise can mask the true effects, e.g., with regard to observed behavior or cognitive processes. In a formal hypothesis-testing framework, this trade-off between resource constraints and statistical significance is best described by the measure of statistical power. Statistical power describes the probability of finding an effect that is actually present in the population of interest. In general, we want our sample size to be large enough to achieve high statistical power while using as few resources as necessary.

To address the challenges in finding a cost-efficient sample size while maintaining high statistical power, researchers can utilize power analysis tools to optimize their study designs. The mlpwr package provides a means to perform simulation-based power analysis for a broad class of applications (Zimmer and Debelak , in press). It fills two previously existing gaps in the literature by allowing for user-defined scenarios with multiple design parameters and explicitly accounting for the cost of study designs during the search algorithm. As Lakens (2022) recently pointed out, there are many power analysis tools available, but learning to use them effectively takes time. In response to this issue, we provide an introduction to the background and the application of the mlpwr package.

This paper is aimed at researchers who want to perform power analysis for complex statistical models, specifically those requiring Monte Carlo simulations. To effectively use the approach outlined in the paper, readers should possess a fundamental understanding of R and be proficient in setting up simulated hypothesis tests, which includes artificial data generation and model fitting.

The remainder of this introduction provides an overview of power analysis for complex study designs, including the motivation behind it, different approaches to conducting power analysis, a brief review of previous research, and an overview of our framework. In the “The mlpwr package” section, we introduce the mlpwr package with a basic example and gradually progress towards more complex usage scenarios. Finally, in the “Practical examples” section, we demonstrate the practical application of the package by presenting two examples based on empirical studies utilizing item response theory and multilevel modeling models.

### Motivation

#### Justifying sample sizes

The recent replication crisis has put low statistical power and replicability of scientific research into focus (Open Science Collaboration , 2015; Button et al. , 2013). Starting from the observation that most published research results might be wrong (Ioannidis , 2005; Simmons et al. , 2011), there have been several developments to improve the replicability of scientific studies (Shrout and Rodgers , 2018). One of these are registered reports, in which research projects are reviewed and conditionally accepted based on sound methodology rather than on the statistical significance of the result. In registered reports, justification of sample size based on power analysis is usually mandatory. For example, in the journal *Nature Human Behaviour*, the sample size should be large enough to achieve at least 95% statistical power (Nature Human Behaviour , 2022). Looking at recent developments, registered reports are indeed accompanied by more frequent justification of sample size (Soderberg et al. , 2021). Another means to ensure replicability is through pre-registrations, where key properties of the planned research are fixed before data collection and statistical analyses. A study by Bakker et al. (2020) found that pre-registered studies had larger sample sizes than earlier psychological studies. However, the study did not find that explicit recommendations for performing power analysis led to larger sample sizes. Although the sample size is still often only stated but not justified (Lakens , 2022), stating and justifying a sample size before conducting a study is arguably becoming common practice.

#### Multiple design parameters

In addition to sample size, a study can be characterized by other adjustable design parameters that influence statistical power. Instead of only one study design parameter (e.g., sample size), a study design can then be characterized by a set of two or more study design parameters. For example, the design parameters may consist of the sample size plus

- the number of measured time points in a longitudinal design,
- the number of questionnaire items,
- the number of trials per participant in an experimental design (see also Baker et al. 2021), or
- the number of groups in a multilevel design.

In these examples, calculating the power and finding a good study design can be much more difficult than if there is only one design parameter.

As an illustrative example, we consider testing the effect of a novel reading exercise on the reading competency of elementary pupils. To ensure the reliability of our results, we want to collect pupils from multiple schools. We set up two conditions that differ in whether an older reading exercise or the novel exercise is used. Within each school, half of the pupils get administered the old exercise and half the new one. We use a multilevel/mixed-effects model to analyze the data and specify a random intercept and slope for the different schools. This is done to control for different baseline reading skills as well as potential differences in the effectiveness of the exercise in the different schools. As a predictor, we include the type of reading exercise (old versus new). The design parameters are a set of the number of schools and the number of pupils per school. We want to find out how many pupils from how many schools are sufficient for our research design to have a power of .95.

**Fig. 1:**
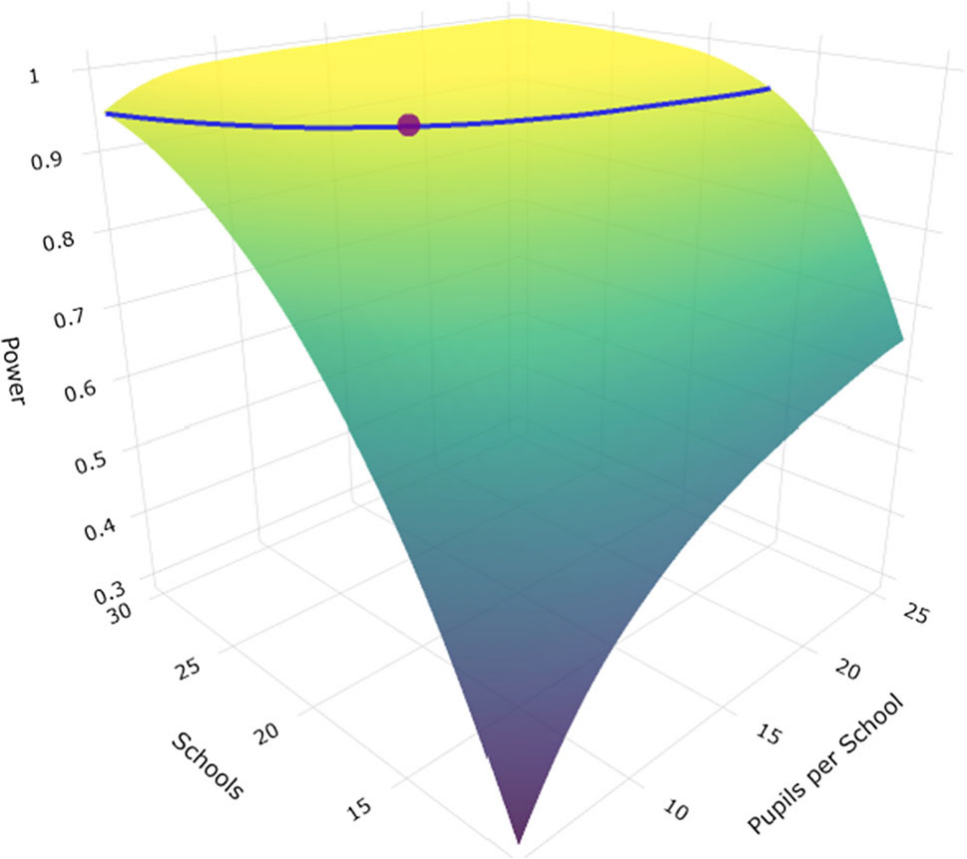
Power for multiple design parameters. The surface displays the power levels for all design parameter sets. The *brighter colors* indicate higher power values, whereas *darker colors* correspond to lower values. *Design sets marked with a line* correspond to a power of .95. The optimal design parameter set when also accounting for costs is marked with a *dot*

Figure 1 shows the power as a function of the number of schools and the number of pupils per school on the *x*- and *y*-axes. The surface shows the power for all sets of design parameters (including designs that are not realistic because they use fractions of pupils or schools). The brighter colors indicate higher power values, while the darker colors indicate lower ones. As we will go into detail below, obtaining this relationship between the design parameters and power can be complicated and computationally intensive, depending on the specific study design and hypothesis test we consider.

As can be seen in Fig. 1, we have multiple options to pick from if we want to choose a set of design parameters that corresponds to a specific power. To help in our example, we have highlighted all sets with a power of .95 with a line. For example, 22 pupils each from 17 schools and 11 pupils each from 21 schools would both correspond to a power of approximately .95. Such design sets are termed power-equivalent in the literature (von Oertzen et al. , 2010). They underline the fact that a power analysis in multiple dimensions is more complex, as there is no simple answer to our question of which design to choose.

#### Costs of study designs

When we need to choose one design, we can usually distinguish between designs that are power-equivalent using the required resources, such as the financial costs to recruit more participants or increase the number of groups.

For our example above, we want to assume that the elementary school students participate without compensation. However, producing one set of exercise material costs $100. We assume that the study is conducted by the same investigators in each school, therefore this exercise material can be reused in all schools. Also, performing the evaluation with another group of pupils in an additional school produces costs of $200. We can express the total costs of our study as follows:

$$\begin{aligned} \text {Cost} = 100 \cdot \text {Pupils per school} + 200 \cdot \text {Schools} \end{aligned}$$

We can use this function to calculate the costs for three promising design parameter sets, see Table 1. If we continue evaluating the costs for all promising candidate sets, we find that only one of the power-equivalent designs on the line in Fig. 1 is optimal with respect to the overall study cost. This design (11 pupils per school and 21 schools) is marked with a dot.

**Table 1:** Sets of design parameters and associated power and cost

Pupils per school	Schools	Power	Cost
22	17	.951	5600
11	21	.952	5300
6	27	.949	6000

Finding such cost-optimal designs in the presence of multiple design parameters can be very computationally intensive with currently available implementations. The mlpwr package we introduce in this paper is aimed to provide an efficient approach that is easily accessible.

Another challenge that can arise in the context of multiple design parameters is that there is a strict limit on resources, such as a maximum budget for a study. In this case, one wants to find a design with maximum power among designs that are similar in terms of cost. For example, if two experimental designs are associated with a cost of $1,000, we would prefer a set of parameters with a power of .9 over a set with a power of .8. The design with a power of .9 might then be the best compromise given the cost constraint. Also for this scenario, the mlpwr package aims to provide an efficient solution.

### Approaches to power analysis

Before we present our implementation to find optimal designs in these scenarios, we want to give an overview over power analysis methodology and implementations. Methodologically, two approaches can be distinguished for determining the power of a study. One is the analytical or formula-based approach. It is generally fast but sometimes unavailable, in particular for more complex or uncommon statistical hypothesis tests (Lakens , 2022). An alternative approach with higher availability but higher computational effort is the simulation-based approach. Both analytical and simulation-based approaches can be used in the presence of multiple design parameters.

#### Analytical approach

**Fig. 2:**
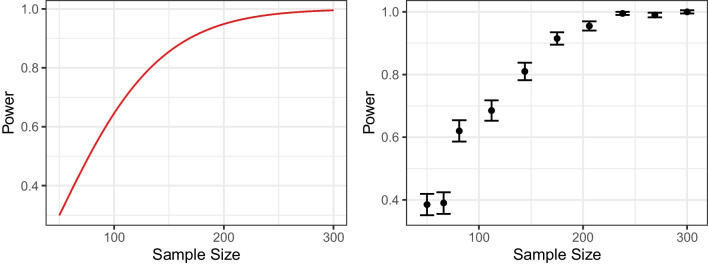
Analytical approach (*left panel*) and simulation-based approach (*right panel*) to power analysis. In the *right panel*, power was estimated using 200 simulations for each sample size. The *error bars* represent the standard error of the power estimate

In analytical approaches, the known mathematical relationship between design parameters and statistical power is the basis for power analysis. This is illustrated in the left panel in Fig. 2. It shows the power for using a dependent *t* test to detect a mean difference between two measurement time points. With such a graph describing the relationship between sample size and power, we can determine the power for a given sets of study design parameters or sets of design parameters with a desired power.

There are well-known implementations of analytical power analysis. Examples include the standalone G*Power software (Faul et al. , 2009) or the pwr package (Champely , 2020) implemented in R (R Core Team , 2022).

The speed of analytical approaches makes them the first choice for simpler models. However, a slight change in the study design and the hypothesis test in question may require a different analytical treatment. This is because it can be difficult or even impossible to derive analytical formulas for more complex models (such as determining the power to test a random effect in a multilevel model, Cools et al. 2008). For this reason, a common challenge is that analytical solutions are unavailable (Lakens , 2022).

#### Simulation-based approach

The simulation-based approach is based on repeated simulation of the desired study. Each simulation run involves generating artificial data, fitting a statistical model, and performing a statistical test. The resulting rate of significant test results across simulation runs serves as an estimate of statistical power. When we perform a simulation-based power analysis, we want to repeat this process several times for different sample sizes or, put more generally, for different sets of study design parameters.

The result of this approach is shown in the right panel of Fig. 2 for our illustrative example. As indicated by the error bars, we receive estimates together with their uncertainty for the power at the different sample sizes. For each sample size, the error bars indicate the standard error of the estimated power. The estimated power for a given sample size may differ from the true power and becomes more accurate if we increase the number of simulation runs. Moreover, compared to an analytical approach, we can only get an approximation of the overall relationship between sample size and power. Determining the optimal sample size, e.g., to achieve a power of .95, can therefore be computationally intensive.

### Guiding simulation-based power analysis

Without using systematic methodology to guide a simulation-based methodology, one can perform a manual search for a suitable set of design parameters. Manual search involves estimating power for a particular set of design parameters and then repeating the procedure for a different set of design parameters based on the result and subjective evaluation. It should be noted that this procedure is much easier if there is one design parameter compared to multiple parameters. For example, if there is only sample size, and the estimated power is too low for a specific sample size, we can continue searching among higher sample sizes. If we consider a set of multiple design parameters, it is much more difficult to decide which design parameter set to try next. As we have seen in Table 1, promising sets can be far apart from each other. In such situations, it can be difficult to find all good candidate sets, even if we have already stumbled across some. Therefore, depending on the computational complexity of individual simulations, a manual search may not be computationally feasible, especially for multiple design parameters. In addition, manual search is not guaranteed to find optimal parameter sets in terms of power or cost. This is because in scenarios with multiple design parameters, we may completely miss the region where the optimal set of design parameters is located during a manual search.

To compensate for these drawbacks, especially when individual simulation runs take a long time, we need additional tools to guide simulation-based power analyses. The goal of these tools is to increase computational efficiency in approximating a set of design parameters with desired power and cost.

#### Basic methods

One basic method to guide a simulation-based power analysis is to systematically search a grid of plausible parameter values. It is commonly applied for study designs with one design parameter and often sufficient in this case. There are many available implementations of grid search for simulation-based power analysis that provide additional support for specific applications. Examples include simr for multilevel models (Green and MacLeod , 2016), mc_power_med for mediation models (Schoemann et al. , 2017), or PowerLAPIM for intensive longitudinal designs (Lafit et al. , 2022). To facilitate the decision for a specific study design, one can repeat the grid search with iteratively smaller grids. As a more standardized alternative, a bisection search algorithm to systematically narrow down on a suitable sample size was suggested by Williams et al. (2007) and Jung (2008).

#### Surrogate modeling

When we consider study designs with multiple design parameters, guiding a simulation-based power analysis becomes more difficult. This is because the power associated with a single design parameter set can take a long time to estimate and the possible number of design parameter sets becomes much larger when there are multiple design parameters. Surrogate modeling represents a straightforward solution to this problem. The idea of surrogate modeling is to approximate a relationship that is very costly to investigate with a function that is cheaper to evaluate (Bhosekar and Ierapetritou , 2018; Forrester and Keane , 2009). One early example application is the relationship between geographic location and groundwater quality (Razavi et al. , 2012). To study this relationship optimally, for example, to find optimal sites for groundwater remediation, we would need to study groundwater quality at countless sites. A more economical option is to test fewer sites and use a surrogate model for the relationship between site and groundwater quality. Using the fitted surrogate model, one can then make predictions about water quality at untested sites based on water quality at neighboring sites. Once a site of interest is identified, one can test the groundwater there to measure quality. Then one can expose the surrogate to this newly collected data and adjust it accordingly. At the end of this iterative process, an optimal site for groundwater remediation can be found, requiring only a small number of actual tests.

Another example of surrogate modeling can be found in psychophysics, where a psychometric function such as a logistic or Weibull function is used to model the relationship between stimulus intensity and perception intensity (Leek , 2001). The function describes the probability of perceiving a stimulus at a certain intensity, and, once fitted, can be used to predict the stimulus intensity at which the perception threshold (i.e., 50% perceived) is reached (Watson and Pelli , 1983).

**Fig. 3:**
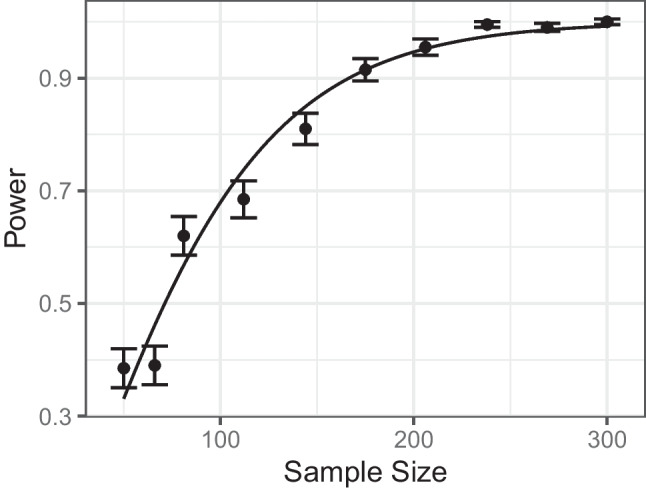
A basic surrogate model fit. The underlying example uses a *t* test to test for a mean difference between two time points. For each sample size, the *error bars *represent the standard error of the estimated power

We can also adopt the idea of surrogate modeling to the functional relationship between study design parameters and power. For the right panel of Fig. 2, we had estimated power for a range of sample sizes. For the purpose of an example, we want to find a sample size that corresponds to a power of .95. We can use logistic regression as a surrogate model to fit this relationship, see Fig. 3. Through this fitted model, we obtain a good idea of the relationship between sample size and power, similar to the known functional relationship in an analytical power analysis. We can use this functional relationship to predict the power for a sample size that we did not perform a simulation at beforehand. For example, for a sample size of $N=100$, the power can be predicted to be .680. We can also obtain a guess for which sample size will imply our desired power of .95. Accordingly, we would next perform further simulations using a sample size of $N=204$. Afterwards, we can fit our surrogate model again to refine our idea of the relationship between sample size and power. In this way, we can iteratively approach a suitable sample size.

Surrogate modeling is computationally more efficient than manual search or grid search. With surrogate modeling, a small number of simulations can be sufficient to obtain an idea of the overall relationship and appropriate design parameter sets. This is particularly pronounced with multiple dimensions of design parameters. Since there are many possible options, it can be very difficult to select the next set of design parameters to estimate performance in a manual search. Also, in a grid search, the computational cost can be very high if we want to estimate the performance for a larger number of design parameter sets. Surrogate modeling can be more efficient than grid search even when there is only one design parameter: In a simple example comparing it to a grid search reported in Green and MacLeod (2016), our approach required only a fifth of the computational effort (Zimmer and Debelak , in press).

### Previous research and implementations

Surrogate modeling in the context of optimizing the power of a study design has already been applied in multiple studies, including clinical trials and network models. In this section, which is aimed more at the technically interested reader, we provide an overview of these.

Mulay et al. (2021) applied surrogate modeling of power in three scenarios: A linear regression, logistic regression, and a repeated measures ANOVA. As design parameters, they varied not only the sample sizes, but also the model parameter weights, e.g., the regression weights. To predict the power as a function of the study design parameters and the model parameter weights, their most successful surrogate model was an approach using a neural network. Presumably because they focused on describing the overall power function, they did not apply an iterative algorithm to find an optimal constellation of study design parameters. To find an optimal design parameter set, the use of an iterative algorithm is usually a better strategy in terms of computational cost. We also use one in the package mlpwr to focus computational resources on finding promising design parameter sets.

Wilson et al. (2020) use surrogate modeling in the context of a clinical trial. They apply a multilevel model and consider multiple study design dimensions. Since their statistical approach has an unknown alpha error, they optimize for a desired alpha error alongside a desired statistical power. They use R to implement their surrogate model algorithm, and find a selection of suitable sets of design parameters. One drawback of the approach is that the cost of the parameter sets is not taken into consideration during the search. This can be computationally suboptimal if many simulations are performed for design parameter sets that are relatively cost-intensive. An approach that takes the cost of design parameter sets into account during the search would be more desirable for computational efficiency. By considering cost, we can discard design parameter sets that have promising power but high cost (such as the third set in Table 1) early during the search.

Another application for clinical trial designs was recently published by Richter et al. (2022). They performed optimization of multiple study design parameters for an adaptive seamless design (Friede et al. , 2020). They considered multiple design parameters, such as a proportion of participants allocated to different stages of the research design, but the total sample was held fixed. For their surrogate model implementation, they used the mlrMBO package (Bischl et al. , 2017). mlrMBO is a flexible and comprehensive implementation of surrogate modeling written in R. In their study, Richter et al. (2022) found that surrogate modelling allows suitable designs to be found in a fraction of the time required by an exhaustive grid search. Because the total sample size was held constant in their example, the cost of the design parameter sets did not need to be considered in the search algorithm. However, this may be desirable in other scenarios where costs vary more between design parameter sets.

Constantin et al. (2021) applied a surrogate modeling approach to find adequate sample sizes for network models. For network models, power usually refers to achieving a desired performance measure, such as a desired sensitivity, rather than significant *p* values. They provide the powerly R package, which is also available on CRAN. It supports optimization in one design parameter dimension and applies monotone splines as a surrogate model. While the package is designed for network models, it can also accommodate a wide range of other statistical models.

### Our framework

In a recent manuscript, we proposed a novel surrogate modeling framework for finding optimal designs (Zimmer and Debelak , in press). Our framework is based on the core ideas of iterative data collection and predictions, as described in the “Surrogate modeling” section, and can be located in the literature alongside existing approaches for modeling statistical power in multiple design parameter dimensions, such as those presented by Wilson et al. (2020) and Richter et al. (2022).

As an implementation of our framework, we developed the mlpwr R package, which can be applied to a wide range of study designs and statistical hypotheses. The package closes two gaps in the literature by providing an implementation for multiple study design parameters and allowing for the consideration of costs during optimization. By taking cost into account, our framework enables a more efficient search for optimal designs compared to other approaches, such as the one presented by Wilson et al. (2020), where a selection by cost can only be performed after identifying suitable design parameter sets.

Our algorithm involves repeated phases of simulated hypothesis tests and the prediction of suitable study designs. In the Appendix, we provide a detailed description of the algorithm and its phases.

In an extensive simulation study, we demonstrated the accuracy of our framework for a range of different scenarios, including *t* tests, ANOVA, item response theory (IRT) models, multilevel models, and multiple imputation (Zimmer and Debelak , in press).

It is important to note that our framework is designed to address a broad class of study design scenarios, which may result in less-than-optimal performance in specific situations. For example, as we take a general function approximation approach to model the relationship between study design and statistical power, there will be less flexible approaches that offer better solutions for specific scenarios. However, by prioritizing good results across different scenarios, we believe that our framework can be a valuable tool for power analysis in practice.

## The mlpwr package

This section introduces how to find optimal study designs using the mlpwr package. To begin, we present an illustrative example to showcase the basic functionality of the package. Next, we delve into more complex usage scenarios and introduce them alongside specific topics, such as setting up simulation functions, terminating and continuing a search, selecting a surrogate model, and managing multiple design dimensions.

The mlpwr package is intended as an easy-to-use interface to performing the surrogate modeling approach to study design finding. Since optimization of power is a very specific use case of surrogate modeling, the mlpwr package provides a specifically tailored interface on top of its own implementation of surrogate modeling. This serves the purpose of simplifying the use of surrogate modeling compared to directly using more general implementations, e.g., mlrMBO.

This tutorial uses version 1.1.0 of the mlpwr package. It is available on CRAN^1^. All code used in this tutorial paper is also available in the supplementary material at https://osf.io/xebsj/.

### An illustrative example

First, we will go through a simple example to show the basic functionality of the package for determining a sample size. We will cover additional functionality and options in the sections from “Simulation function” to “Additional options”. We therefore consider the evaluation of a medical intervention to reduce the symptoms of a cold. Our goal is to determine a suitable sample size for a study that examines the utility of the intervention. Specifically, we want to test whether the within-person difference in symptoms of a cold between two time points (before and after an intervention) differs from 0. For the sake of simplicity, we assume that the cold symptomatic is measured using real values. We furthermore assume that the intervention has a small effect size of Cohen’s $d = 0.3$ and apply Student’s *t* test within subjects using an alpha level of .01.

As a prerequisite to the surrogate modeling procedure, we express a single simulation run in a simulation function simfun_cold. It takes the study design as input and outputs an indication of significance:
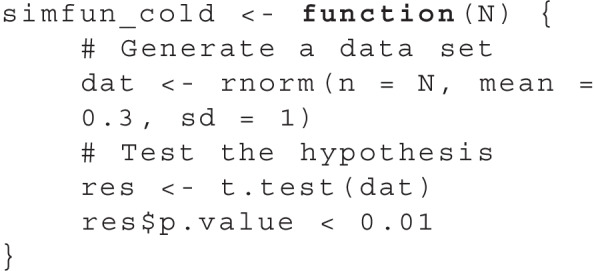


In the simulation function, the object dat, which corresponds to artificial data, is first generated based on the sample size N. This data set contains the reduction of cold symptoms between the two time points for each person in the study. We then perform the planned *t* test to test whether the reduction of cold symptoms differ from 0, and obtain a *p* value. Finally, the output of simfun_cold is either TRUE if the *p* value is less than .01 and FALSE otherwise. To obtain an estimate of the statistical power for a sample size *N* = 120, we can repeatedly execute simfun_cold(N = 120) and obtain the rate of successful tests. For further guidance on setting up a simulation function in more advanced settings, see the “Simulation function” section.

We can now use the simulation function simfun_cold as an input to the find.design function in mlpwr to perform the search using surrogate modeling. As boundaries to search within, we want to specify 100 participants as a lower bound and 300 participants as an upper bound. We can do this by specifying the vector c(100, 300) in the boundaries argument. We could also put this vector inside a list, specifying that the boundaries refer to the sample size n: list(n = c(100, 300)). This will be more useful later when we consider multiple design parameters. Furthermore, we indicate that we search for the sample size that corresponds to a power of .95.
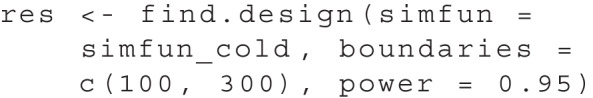


We can display a summary of the results with summary(res): 
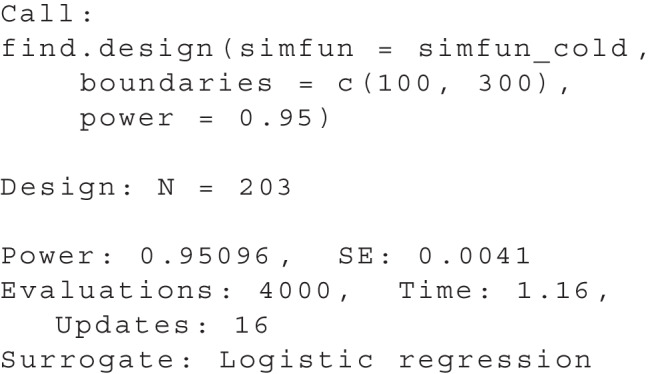


In the console output, we can read off the sample size predicted by the algorithm, as well as the estimated power and estimated uncertainty (standard error, SE) for this design. In this example, the final predicted sample size was $N=203$ and the power was estimated to be .95096 for this sample size ($SE = 0.0041$). The summary also provides further information about the search. Accordingly, the simulation function was evaluated 4000 times, the calculation took 1.16 s in total, and the updating phase was performed 16 times. During each updating phase, the simulation function is evaluated at promising locations. Technical details such as the number of evaluations during each update and more information about the different algorithm phases are described in the Appendix. A logistic regression was used here as the surrogate model. We will go over the surrogate models and the available options in more detail in the “Surrogate models” section.

**Fig. 4:**
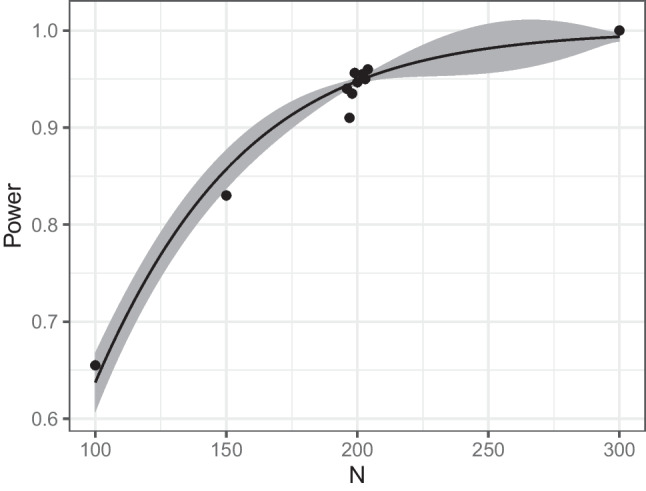
Illustrative example: graphical representation of the result

We can obtain a graphical representation of the result by using plot(res), see Fig. 4. The *black dots* in this plot indicate the estimated power for all simulated sample sizes. The *gray ribbon* shows the estimated standard error for all sample sizes within the boundaries (see the “Surrogate models” section for more details on its calculation). The graph illustrates the efficiency of the surrogate modeling method, namely that the algorithm leads to a concentration of search effort in the promising region. This can be seen from the fact that many of the simulated data points are close to the optimal value around $N=200$. This speeds up the search considerably, especially for more complicated designs, as we will see below

### Simulation function

The simulation function requires the study design parameters as input and returns an indication of significance. While this allows for a high degree of flexibility as many simulation functions are compatible, depending on the study design being planned, setting up the simulation function correctly can of course be challenging. Most often, the simulation function internally consists of two steps, as exemplified by the simfun_cold above.

1. Generating a data set. This can be done using an object that contains a fitted model or via specifying the model parameters directly.
2. Testing the hypothesis. This usually first involves fitting a statistical model to the generated data. Using the model, we can perform a statistical test and output the significance.

In most cases, the test of the hypothesis is more straightforward for applied researchers, as it is a standard use case of R packages and taught in many statistics courses. Generation of artificial data is however less often practiced and may be unfamiliar to applied researchers. An important prerequisite for generating data and planning a study design in general is the determination of the expected size of the effect to be studied (Lakens , 2022). There are many definitions of effect size that depend heavily on the statistical model used (e.g., Brysbaert and Stevens 2018; Lorah 2018; Chalmers 2022). Options to determine an effect size include using the results of a meta-analysis, consulting with experts, or conducting a pilot study. To assist with data generation, many R packages offer special functions that can greatly help with this step. Examples are the simulate function in the lme4 package (Bates et al. , 2015) and the simdata function in the mirt package (Chalmers , 2012). These functions can be used as a part of simulation functions to generate a data set with the desired study design parameters, such as a desired sample size. Because of this possibility, the mlpwr package is highly compatible with any existing artificial data generation functions in other packages and can be used in combination with them.

To give some examples of how to set up simulation functions, including using data generation functions from other packages, we provide the vignette simulation_functions.Rmd^2^. Here, we collect different example simulation functions. These are fully functional simulation functions that can serve as a blueprint and starting point for adaptation to your own use case. The amount of customization required may vary depending on your planned scenario of data generation and hypothesis testing. The included templates are from the following areas and use the respective R packages:

- *t* test, ANOVA, and generalized linear models using the stats package (R Core Team , 2022)
- Item response theory models using the mirt package (Chalmers , 2012)
- Multilevel models using the lme4 package (Bates et al. , 2015)

### Terminate and continue

In the course of the surrogate modeling algorithm, many evaluations of the simulation function are performed to obtain power estimates. By default, find.design terminates after 4000 evaluations of the simfun. To change this according to your needs, you can set the evaluations argument of the function to a custom value. Another option for termination is reaching a specified certainty about the estimated power for the predicted study design. Using ci = .03, for example, we can indicate that we want to reach a 95% confidence interval with a width of .03 or smaller for the power estimate. Finally, we can specify a number of seconds after which the function should terminate via the time argument. We can set multiple criteria for termination, in which case the algorithm ends if at least one of them is met. For example:
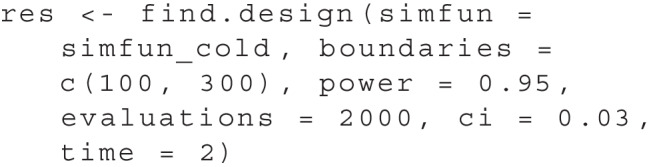


In this case, the algorithm would terminate when either at least 2000 evaluations of the simulation function have been performed, the estimated confidence interval width is less than .03, or more than two seconds have elapsed.

In case the result found by find.design should still be improved further, you can continue a terminated search at any time, e.g., if more evaluations should be performed or the confidence interval width should be even smaller. The simplest way to do so is via:
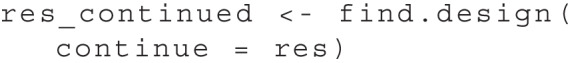


This way, the algorithm is continued using all previously performed simulation function evaluations. You do have the option to specify different termination criteria than before at this point. If not, all previously specified options are retained (e.g., time, ci, evaluations). One use case would be to first run the algorithm for one minute with time = 60 and then continue the search for five minutes with time = 300.

### Surrogate models

During the search, the surrogate models are used to fit the relationship of design parameters and power to determine the next search location, see Figs. 3 and 4. By default, find.design uses a logistic regression when one design dimension is used (e.g., when only sample size is searched for) and Gaussian process regression (GPR) when multiple design dimensions are used (e.g., in multilevel models when sample size and the number of clusters are searched for). Technically speaking, in GPR, the design parameter space is modelled as a collection of random variables (Rasmussen and Williams , 2006). The relationship of the estimates of neighboring points is described through a specific covariance function, which functions as a prior. This is one of the major advantages of GPR: One can estimate not only the power but also the error variance of sets of design parameters that have not been simulated yet. We make use of this property to create the gray ribbon in Fig. 4 of the illustrative example. The variance estimated using GPR is used to gain more insight into the collected data by showing which ranges of sample sizes have been closely investigated and which remain unexplored. It is important to note that this gray ribbon is always calculated using GPR, even if the line in the plot represents a different fitted surrogate model (e.g., logistic regression). As an alternative to GPR, one may use support vector regression (SVR). Based on more general work on surrogate modeling, SVR can be expected to perform better than GPR for higher numbers of design parameter dimensions (Jia et al. , 2020). However, as SVR generally takes longer than GPR and offers similar performance in the simulation studies in Zimmer and Debelak (in press), we recommend leaving the surrogate model settings at their default. Nevertheless, based on future research, specific surrogate models may emerge as beneficial for specific scenarios, and the default surrogate models could be modified.

The surrogate models can be chosen via the surrogate argument, for example by:
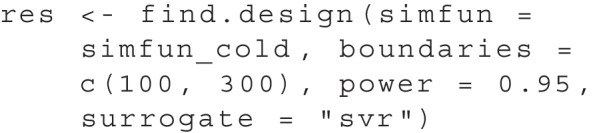

**Fig. 5:**
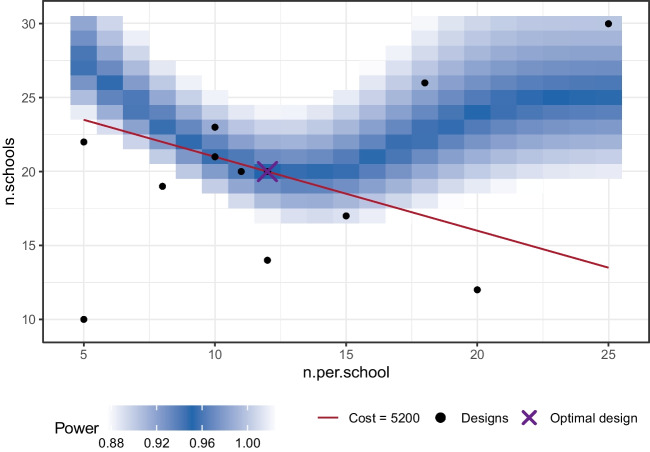
Multiple design dimensions: graphical representation of the result

### Multiple design dimensions

We turn again to our example in the Introduction (“Multiple design parameters” section), where we wanted to evaluate a reading exercise and tried to find adequate numbers of pupils per school n.per.school and number of schools n.schools. We apply a multilevel model and test the new versus an old reading exercise as a fixed effect. To account for a potentially different effectiveness of the reading exercise among the schools, we include the schools via a random intercept and slope. In the interest of brevity, we include the simulation function simfun_multilevel in the file papercode.Rmd in the online supplement. It has the two arguments n.per.school and n.schools and outputs either TRUE or FALSE depending on the significance of the hypothesis test.

As we observed in Fig. 1 and Table 1, there are multiple design parameter sets that correspond to a power near .95. To differentiate among those, we assumed that each exercise material costs $100 and every additional school implies additional costs of $200. We can express the overall cost of the study with a cost function in R:
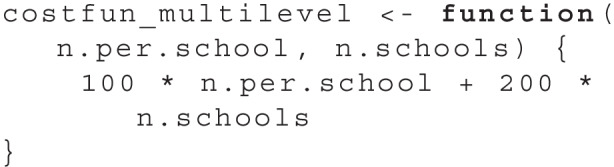


We use both the simulation function and the cost function as arguments in the find.design function to start the search. Again, we need to specify the boundaries for the design parameters. For multiple dimensions, we need to specify the boundaries as a list, with one element for each design parameter dimension:
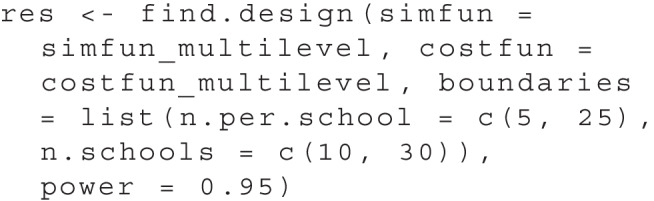


The output of summary(res) now shows:
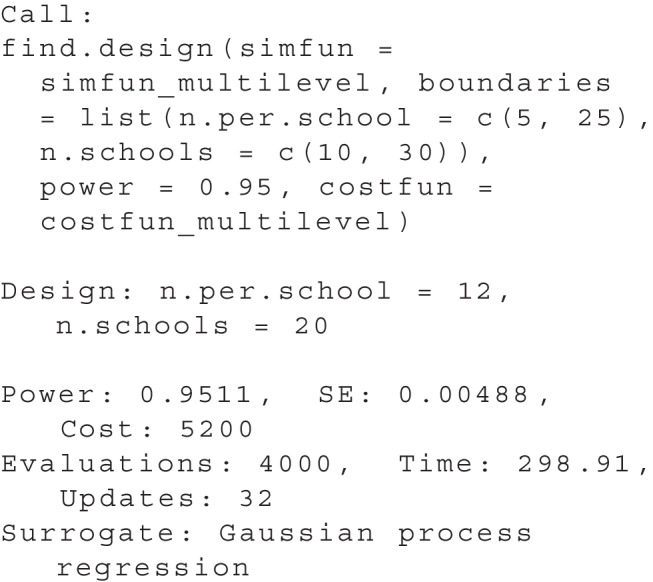


The result shows that the optimal design is to test 12 pupils in each of a total of 20 schools. This design will produce a total cost of $5,200. All further output is identical to the one-dimensional use case.

To visualize the result, plot(res) produces a two-dimensional plot, see Fig. 5. Here, we can see all design parameter sets for which simulations have been performed as *black dots*. A purple ’X’ highlights the optimal design and a *red line* indicates all parameter sets that have the same cost as the optimal design. Additionally, a heat map illustrates the power as estimated by the surrogate model: Design parameter sets with a power closer to the desired power are shown in a *darker blue*.

**Fig. 6:**
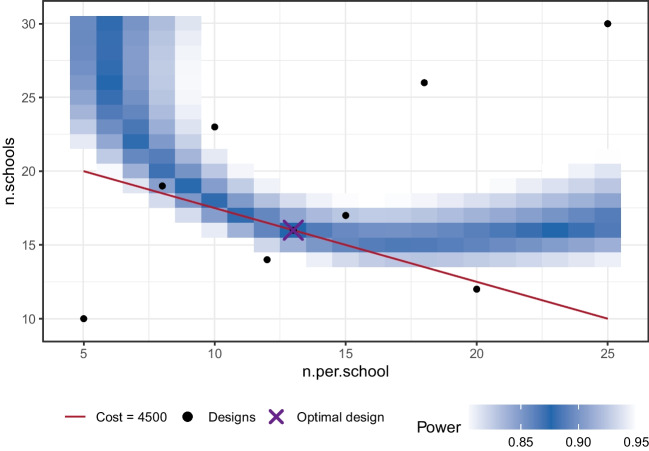
Multiple design dimensions: search considering a cost threshold

As mentioned above, another usage scenario than reaching a desired power is to reach a maximum power given a cost threshold. This can be relevant when there is a limit to the available resources, e.g., a maximum grant budget. The search for a design can then be performed by replacing the power argument with a cost argument. For example, to search the set with the highest power among all sets that involve a cost of $4,500 or less, we can use:
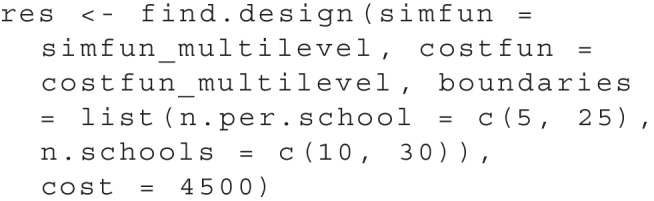


Knowing that the optimum for a power of .95 went along with a cost of $5,300, we can expect that the power of the optimal value is lower for this scenario. The output of the find.design function can be viewed using summary(res):
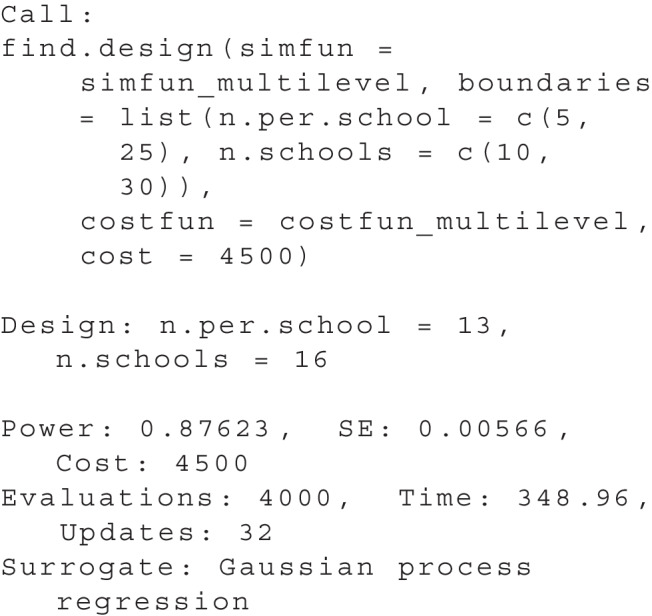


The resulting plot is shown in Fig. 6. From this plot, we can confirm that the optimal design has a power of about .88. Please note it is also possible to find optimal designs with more than two design dimensions, see the vignette extensions.Rmd.

### Additional options

One set of additional options concerns the specifics of the surrogate modeling algorithm. The initialization phase of the algorithm, to which the following arguments refer, and the other phases of the algorithm are explained in more detail in the Appendix. Additional options include the number of sets of design parameters (n.startsets, default is 4), or the percentage of total evaluations used during initialization (init.perc, default is 20%). These are available for experimentation or for when the default settings should not lead to satisfying results.

For example, increasing the percentage of evaluations or the number of points during initialization can be useful if the surrogate model initially fails to capture the shape of the power function. To know if this was the case, one can find the number of failed surrogate model fits using res$n.bad.fits, given that res was created by the find.design function.

In case the evaluations of the simulation function take longer, it might be advisable to backup intermediate results to prevent the loss of data. This can be done by indicating a directory to save the generated files at using the autosave_dir argument.

To investigate the simulated data during the algorithm in more detail, you can use the simulations_data function. It outputs a data frame that, for each set of design parameters, includes the cost, the estimated power and SE, the surrogate model estimated power and SE, and the number of performed evaluations. Here, the power is estimated using the proportion of significant hypothesis tests among the performed evaluations. The SE is estimated using $\sqrt{p(1-p)/n}$, where *p* is the estimated power and *n* is the number of performed evaluations. It should be noted that, for the estimation of SE using a surrogate model, GPR is employed because it is capable of variance estimation, unlike the other surrogate models.

To allow more experimentation, we have included the possibility to change the options of the surrogate models. This can be done via the control argument of the find.design function. All arguments specified here are passed on to the respective model function. One example for GPR is to specify the above mentioned covariance function by setting control = list(covtype = "gauss").

## Practical examples

We further demonstrate the package with two practical examples inspired by recent studies. The first study set out to investigate fairness in a test for scientific reasoning and applied IRT models (Opitz et al. , 2021). Based on the original data set, we determine a suitable sample size to achieve a power of .95. The second study evaluated an intervention to increase psychological resilience in an international sample and applied a multilevel modeling approach (Wang and Rhemtulla , 2021). Again based on the original data, we determine the number of countries and participants that would be needed for a replication study.

### Example 1: Item response theory

A common challenge in developing educational tests such as PISA (OECD , 2017) is ensuring fairness. One factor that can lead to unfairness in a test is when certain groups have advantages they should not have. An example for PISA would be if an item is less difficult in one language version than in another. For example, English-speaking participants may have an unfair advantage in this case. When the same item can be easier or more difficult in different language versions, this is referred to as differential item functioning (DIF) in the IRT literature.

Opitz et al. (2021) investigated DIF for a test of scientific reasoning. They hypothesized that some specific items might be easier for individuals with a physics degree than for individuals with a biology degree or vice versa. One possible explanation is that some items do not exclusively measure scientific thinking but also benefit from domain-specific knowledge.

We want to determine the sample size for a scenario based on the original study by Opitz et al. (2021). The data used in this example are included in their published article. We first divide the participants into two groups, depending on whether they study "physics" or "other" subjects. Then, using the Rasch model (Rasch , 1960), we estimate item parameters separately for both groups, using the original, publicly available data set. Using these item parameters, we set up a simulation function simfun_irt to generate artificial data in the form of responses to the scientific reasoning test. The simulation function then performs a test for DIF and returns the result. It is included in the file papercode.Rmd in the online supplement for the interest of brevity. In this particular case, we use a score-based test by Strobl et al. (2015) that detects whether DIF is present in any items. Alternative approaches could also be used, including those that measure DIF only for specific items.

**Fig. 7:**
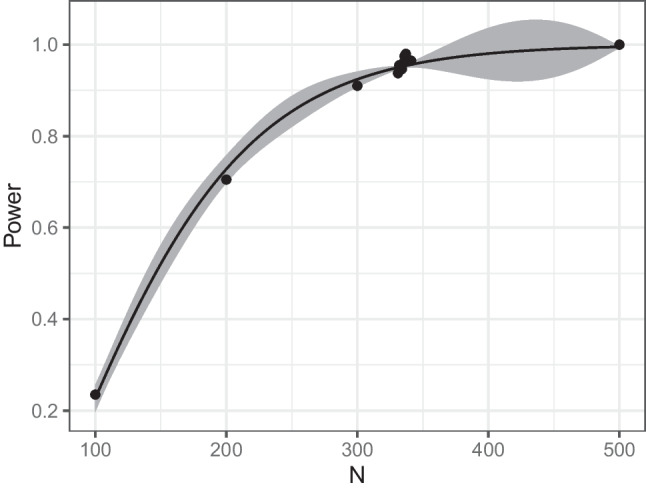
Example 1: Resulting relationship of sample size and power

These prerequisites allow us to use the find.design function to determine a suitable sample size as introduced in the section “The mlpwr package”.
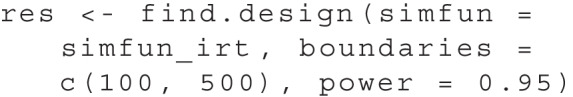


summary(res) gives us the following output:
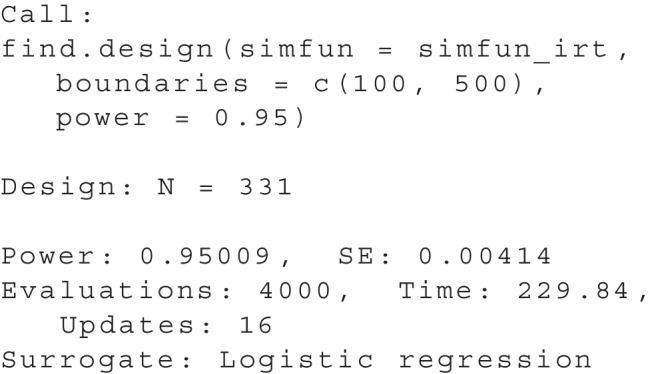


The approach leads to a sample size of $N = 331$ with a high confidence of the implied power, as $SE = 0.00414$. We can obtain a plot using plot(res), see Fig. 7. To put these results in context, a total sample size of 331 would be required to reliably detect differential item functioning for physics students in comparison with students in other disciplines.

Following this example, the mlpwr package can be used in combination with many other IRT models. To assist in setting up simulation functions, artificial data generation functions are available, e.g., in the R packages eRm (Mair and Hatzinger , 2007) or mirt (Chalmers , 2012).

**Fig. 8:**
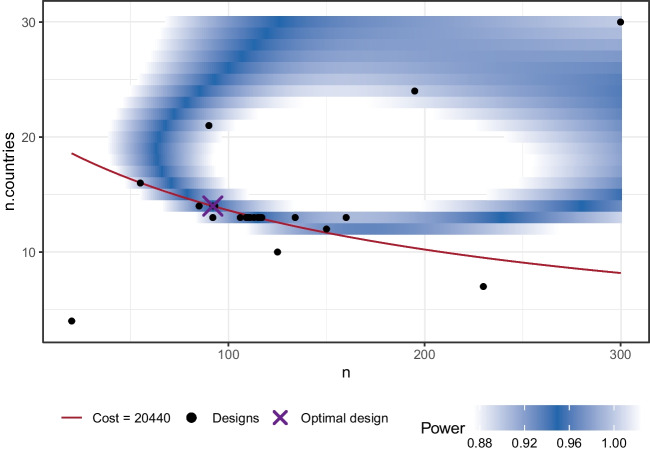
Example 2: Result plot

### Example 2: Multilevel model

To showcase an application to multiple design parameter dimensions, we look at a recent study by Wang and Rhemtulla (2021) that applies multilevel modeling. The data used in this example are included in their published article. In light of the COVID-19 pandemic, the authors investigate brief reappraisal as an intervention to increase psychological resilience. Cognitive reappraisal is a strategy of changing one’s thoughts about a situation in order to influence an emotional response (McRae and Gross , 2020). One exploratory analysis in the work of Wang and Rhemtulla (2021) is directed at the potential mitigating effect of reappraisal on negative feelings. They employed a between-subjects design with participants from 87 different countries. For the statistical analysis, they tested for the fixed effect of the reappraisal intervention against a control condition. In their multilevel model, they included the participant’s country via a random intercept. Furthermore, they controlled for the negative emotion at baseline by including it as another fixed effect. Since this analysis was exploratory in nature, one question for a confirmatory analysis would be: How many participants from how many countries are sufficient to replicate the effect? We assume here that the effect size is actually at least as large as the one found in the original study.

The two design parameters we consider are the number of participants per country n and the number of countries n.countries. As a first step in the simulation function simfun_multi we generate artificial data using all parameter values as estimated from the original data. Then, we apply the same hypothesis test as in the original study. Full code for the simfun_multi is included in the file papercode.Rmd in the online appendix. To again differentiate between power-equivalent sets, we assume in this case that each participant produces a cost of $5 while adding another country costs $1,000. Since n denotes the number of participants per country, the total number of participants is calculated as n * n.countries. We specify a cost function accordingly:
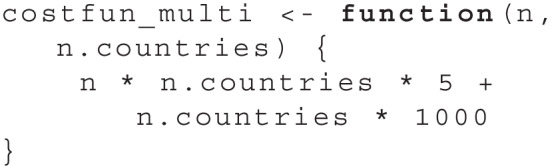


As far as the search area, we choose values for n between 20 and 300 and values of n.countries between 4 and 30. Since there is a large total number of possible sets of design parameters in this case, we set the evaluations to a higher number of 6000. Using these prerequisites, we can perform the search using find.design:
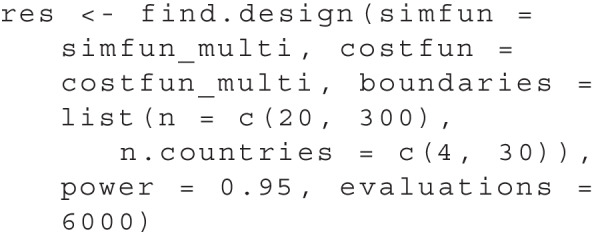


The results can be accessed using plot(res), see Fig. 8, and summary(res):
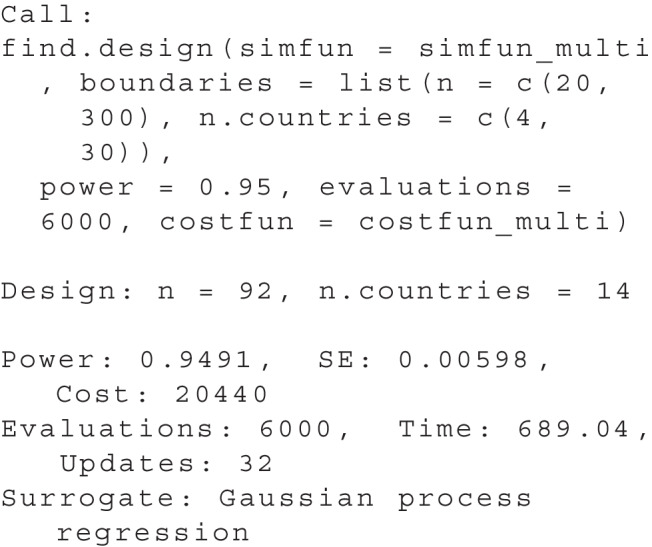


Accordingly, the optimal design for a replication effort with a power of .95 would be to collect data from 14 countries and 92 participants each. The total cost of the study can be estimated at $20,440. It is also apparent from the plot that there are suitable alternative designs (with a similar cost and power) in which data are to be collected from a smaller number of countries (e.g., 13) with a larger number of participants per country (greater than 100).

## Discussion

In this paper, we provided a tutorial for performing sample size planning and power analysis using the mlpwr package. It uses a surrogate model framework that can efficiently organize the simulation-based approach to power analysis. Incremental to already available approaches, the mlpwr package implements the surrogate model approach for when there are multiple study design parameters. Also, it contributes a consideration of costs during the search via a cost function.

While other tools for simulation-based power analysis are tailored more towards specific models, such as simr for multilevel models (Green and MacLeod , 2016) and powerly for network models (Constantin et al. , 2021), the mlpwr package is designed as a general tool. One advantage of this approach is that it is compatible with a large number of study designs, namely those for which simulations can be expressed via a simulation function. The requirements for this simulation function are only that it takes the design parameters as input and returns an indication of significance. As a disadvantage, depending on the context, it can take more knowledge and effort to correctly set up this function. Depending on the desired model, there are many parameters that must be specified for generating artificial data or fitting a model. However, there is already ample work aimed at supporting the preparation of data simulation, such as that of DeBruine and Barr (2021) for multilevel models, as this can also facilitate the understanding of the model. As our own contribution to assist in the definition of simulation functions, we provide the vignette simulation_functions.Rmd. It includes templates for several models, including *t* test, generalized linear models, multilevel models, and item response theory models. We plan to update it regularly in the future, for example to include structural equation models estimated using the lavaan package (Rosseel , 2012).

One possible future extension of the package is the inclusion of additional surrogate models. For example, we could use functions that describe the relationship between design size and power in analytical power analysis as a surrogate model. While these functions may provide more accurate results when applied to corresponding simulation functions, their performance may not be as good as a general function approximation approach, such as Gaussian process regression, across a wide range of scenarios. Our main goal is to offer an approach that works well in many different applications. However, we plan to allow users to input custom surrogate models in the future as our package is currently under development, so they can use specific surrogate functions that may work better for their desired scenarios.

As study designs and statistical methods become more complex, analytical solutions for power analysis are not always available. In addition, the requirements for justifying a choice of design parameters have arguably increased since the replication crisis. With the mlpwr package, we aim to assist in meeting these increased quality requirements for study design planning.

**Fig. 9:**
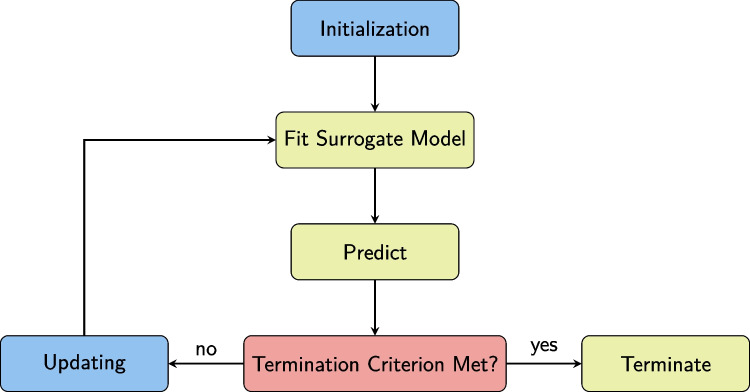
Surrogate modeling algorithm

## Appendix

We describe the surrogate modeling algorithm used in the mlpwr package in more detail. The algorithm is started using the find.design function. The goal of the algorithm is to find a suitable study design, this can be either:

- A design that implies a desired statistical power while going along with minimal costs. Here, the power argument must be specified to indicate the desired power.
- A design that meets a specified cost threshold while maximizing statistical power. In this case, the argument cost must be provided to define the cost threshold.

In addition to the desired power or the cost threshold, the inputs required are the simulation function simfun and the upper and lower bounds for each design parameter. Using these inputs, the algorithm shown in Fig. 9 organizes the search procedure. The algorithm proceeds through several phases, each of which involves a distinct set of operations. These phases are designed to iteratively explore the design space and identify promising regions for further investigation. In this section, we will describe each of the phases in detail, including the specific operations involved and their role in the overall search process.

### Initialization

To enable the algorithm to capture the overall relationship between study designs and power, we begin by estimating power for a select few designs. This data provides the algorithm with a preliminary understanding of where to direct its focus. In particular, certain regions within the design parameter space may exhibit power values that are significantly different from the desired power, while others may be comparatively closer to it. With this information, the algorithm can then proceed to explore the parameter space in a more focused and efficient manner, ultimately identifying the study design that best meets the desired power or cost criteria. We use the quasi-random Halton sequence (Halton , 1960) to select these initial designs. The Halton sequence generates evenly distributed points in a deterministic way, which helps to efficiently cover the design parameter space. In contrast, using random sampling as an alternative could result in points that are clustered together and do not effectively cover the design parameter space. The number of sets used in this phase is determined by the n.startsets argument. It is set to 4 per design parameter dimension as a default. For estimating the power at these design sets, 20% of the total number of evaluations are used. This can be changed via the init.perc argument.

### Fit surrogate model

The function specified via the surrogate argument is fit to all data available up until this point. The defaults are logistic regression ("logreg") and Gaussian process regression ("gpr") for one and two dimensions, respectively. Currently available alternatives are linear regression ("reg") and support vector regression ("svr"). Further details on the available surrogate model options are provided in the “Surrogate models” section. In our paper, we also discuss these options and present a simulation study that informed our selection of default models (Zimmer and Debelak , in press). Given the wide range of potential power functions resulting from the simulation functions, we assume that there is no single surrogate model that would outperform all others in all scenarios.

### Predict

Using the fitted surrogate model, we aim to derive a prediction for a study design suitable for further search. Depending on the scenario (characterized by a desired power or a cost threshold), we search for suitable values. In the case of a design that has a desired power, we do not want to penalize values that have a higher than desired power. Also, we do not want to overlook values that apparently have a suboptimal power, but have been estimated with a relatively high standard error. Simultaneously, we want to favor values with low cost. Analogously, in the cost threshold scenario, we do not want to penalize values with lower than desired cost and we simultaneously want to prefer values with higher power. The functions that are minimized to realize these purposes are in detail:

$$\begin{aligned} \begin{aligned} \text {Desired power: }f_1(x)&= W_1 \cdot \text {relu}\Big (p_g-p(x)-W_2 \cdot SE_p(x)\Big )+\frac{c(x)}{c_m},\\ \text {Cost threshold: }f_2(x)&= W_1 \cdot \text {relu}\Big (\frac{c(x)-c_g}{c_g}\Big )-p(x) \end{aligned} \end{aligned}$$

The relu (rectified linear unit) function maps all negative values to 0 and is otherwise the identity function. Furthermore, *p*(*x*) denotes the power and $SE_p(x)$ denotes the *SE* for the design parameter set *x*, as given by the fitted surrogate model. *c*(*x*) is the cost of the design parameter set *x*. $p_g$ and $c_g$, respectively, denote the desired power and the cost threshold. $c_m$ is the cost of a parameter set in the center of the specified boundaries for the purpose of setting *c*(*x*) in relation. $W_1$ and $W_2$ are weighting constants that balance the two summands in the formulas.

The relu function plays a key role in both functions to achieve our search goals. As long as the argument to the relu function is negative, we don’t apply a penalty for a design parameter set. For example, for the desired power scenario, this may be the case if the power *p*(*x*) is higher than the desired power $p_g$, or if it is slightly lower but the uncertainty $SE_p(x)$ is high. To find the design parameter set that is a minimum to these functions in both cases, we use an evolutionary search algorithm implemented in the rgenoud package (Mebane and Sekhon , 2011). It is inspired by natural selection and seeks to find the global optimum of a function by iteratively generating and selecting candidate solutions based on their fitness.

### Termination criterion met?

We check whether any of the termination criteria is met. This can be the number of evaluations (evaluations), the time passed (time), or the 95% confidence interval of the power for the predicted design parameter set (ci), see also the “Terminate and continue” section. Independent of the specified surrogate model, the latter is calculated using the standard error estimated with Gaussian process regression. Gaussian process regression is used at this point because it can estimate the standard error also for points for which the power has not yet been estimated.

### Updating

The simfun is evaluated at the predicted study design. The number of evaluations used is the same as is used for a single design parameter set during the initialization phase. For example, if the design has one parameter and all settings are set to the default values, this is 200 evaluations.

### Terminate

Before saving the result, the standard error at the final prediction is estimated again, using Gaussian process regression if necessary. After termination, the algorithm can be continued, see also the “Terminate and continue” section. In this case, the algorithm starts in the ’Fit surrogate model’ phase, using all previously generated data as the initialization data.

## Footnotes

1 install.packages("mlpwr") can be used to install the package. It can be loaded using library(mlpwr).

2 Vignettes contain additional guidance for packages. This vignette can be retrieved at https://cran.r-project.org/package=mlpwr or using browseVignettes("mlpwr") after installing the mlpwr package.

## Declarations

### Open Practices Statement

The R syntax for this study is available at the Open Science Framework at https://osf.io/xebsj/. All R packages used in this study are available on CRAN. No experiments were conducted and therefore no experiment was preregistered in the course of this research.
