# Publisher Correction: Sample size planning for complex study designs: A tutorial for the mlpwr package

**DOI:** 10.3758/s13428-023-02312-0

**Published:** 2023-12-13

**Authors:** Felix Zimmer, Mirka Henninger, Rudolf Debelak

**Affiliations:** Psychological Methods, Evaluation and Statistics, Department of Psychology, University of Zurich, Binzmuehlestrasse 14, Box 27, 8050 Zurich, Switzerland

**Publisher Correction: Behavior Research Methods**

10.3758/s13428-023-02269-0

The original online version of this article was revised: a typographical error was not corrected as requested by the author and was left in the published version of the article.

